# Vancomycin Activates σ^B^ in Vancomycin-Resistant *Staphylococcus aureus* Resulting in the Enhancement of Cytotoxicity

**DOI:** 10.1371/journal.pone.0024472

**Published:** 2011-09-02

**Authors:** Hong-Yi Chen, Chien-Cheng Chen, Chun-Sheng Fang, Yi-Ting Hsieh, Mei-Hui Lin, Jwu-Ching Shu

**Affiliations:** 1 Department of Medical Biotechnology and Laboratory Science, Chang Gung University, Taoyuan, Taiwan; 2 Department of Biotechnology, National Kaohsiung Normal University, Kaohsiung, Taiwan; 3 Research Center for Pathogenic Bacteria, Chang Gung University, Taoyuan, Taiwan; Duke University Medical Center, United States of America

## Abstract

The alternative transcription factor σ^B^ is responsible for transcription in *Staphylococcus aureus* during the stress response. Many virulence-associated genes are directly or indirectly regulated by σ^B^. We hypothesized that treatment with antibiotics may act as an environmental stressor that induces σ^B^ activity in antibiotic-resistant strains. Several antibiotics with distinct modes of action, including ampicillin (12 µg/ml), vancomycin (16 or 32 µg/ml), chloramphenicol (15 µg/ml), ciprofloxacin (0.25 µg/ml), and sulfamethoxazole/trimethoprim (SXT, 0.8 µg/ml), were investigated for their ability to activate this transcription factor. We were especially interested in the stress response in vancomycin-resistant *S. aureus* (VRSA) strains treated with vancomycin. The transcription levels of selected genes associated with virulence were also measured. Real-time quantitative reverse transcription PCR was employed to evaluate gene transcription levels. Contact hemolytic and cytotoxicity assays were used to evaluate cell damage following antibiotic treatment. Antibiotics that target the cell wall (vancomycin and ampicillin) and SXT induced σ^B^ activity in VRSA strains. Expression of σ^B^-regulated virulence genes, including *hla* and *fnbA*, was associated with the vancomycin-induced σ^B^ activity in VRSA strains and the increase in cytotoxicity upon vancomycin treatment. These effects were not observed in the *sigB*-deficient strain but were observed in the complemented strain. We demonstrate that sub-minimum inhibitory concentration (sub-MIC) levels of antibiotics act as environmental stressors and activate the stress response sigma factor, σ^B^. The improper use of antibiotics may alter the expression of virulence factors through the activation of σ^B^ in drug-resistant strains of *S. aureus* and lead to worse clinical outcomes.

## Introduction

Vancomycin is used to treat health care-associated infections resulting from multidrug-resistant *S. aureus*, including methicillin-resistant *S. aureus* (MRSA). The first clinical case of vancomycin-intermediate *S. aureus* (VISA) was reported in 1997, followed by the appearance of the first *vanA*-mediated vancomycin-resistant *S. aureus* (VRSA) clinical isolate in 2002 [1]–[3]. A recent study in Taiwan indicated that 2.9% of the MRSA strains isolated were VISA [4].

We are interested in studying the role of an antibiotic when applied to a resistant strain of bacteria. Such an antibiotic may no longer be lethal (or inhibit growth) but instead acts as a stimulus (stress) to drug-resistant strains. If so, whether this type of stress activates σ^B^ and thereby affects the expression of downstream virulence factors remains to be determined. σ^B^ is the alternative σ factor that modulates the general stress response in certain Gram-positive bacteria, including *Bacillus subtilis*, *Listeria monocytogenes*, and *S. aureus* [5]. In addition to the general stress response, σ^B^ regulates cell wall metabolism, membrane transport processes and virulence in *S. aureus* [6], [7]. It has also been shown that virulence factors, such as α-hemolysin (encoded by *hla*) and fibronectin-binding protein A (encoded by *fnbA*), are likely controlled by σ^B^ through the global regulator SarA and/or the *agr* locus [8], [9]. Recently, it was demonstrated that σ^B^ plays a role in central venous catheter (CVC)-associated infections [10].

Concentrations of antibiotics below the minimum inhibitory concentration (MIC) are known to promote a variety of bacterial properties, such as the alternation of virulence; the effects of sub-MICs do not necessarily involve a reduction in the growth rate [11]. It has been reported that sub-MICs of β-lactam antibiotics induce the expression of virulence-associated exotoxin genes in *S. aureus* [12]. In *Clostridium difficile*, another opportunistic pathogenic Gram-positive bacterium, the transcription of major virulence factor genes is induced by sub-MICs of antibiotics [13]. However, sub-MICs of protein synthesis-suppressing antibiotics, such as clindamycin, linezolid and quinupristin/dalfopristin, have been shown to inhibit virulence factors released by *S. aureus* [14]–[16].

In the present study, we hypothesized that σ^B^ may respond to antibiotic stress and mediate the stress signal to downstream virulence factors in antibiotic-resistant *S. aureus* strains. Antibiotics with distinct modes of action were used to investigate their potential in activating σ^B^. The drugs used in this study were ampicillin β-lactam; targets the cell wall), vancomycin (glycopeptide; targets the cell wall), chloramphenicol (suppresses protein synthesis), ciprofloxacin (suppresses DNA synthesis), and SXT (interferes with folic acid metabolism). Because vancomycin is used to treat infections caused by MRSA, the effect of vancomycin treatment on cytotoxicity and the expression of selected virulence genes was tested in VRSA strains.

## Materials and Methods

### Bacterial strains, plasmids and growth conditions

The bacterial strains, plasmids and primers used in this study are listed in Table 1 and Table 2. All bacterial strains were routinely cultured at 37°C with the specific required antibiotics (Sigma) in BHI broth (for *S. aureus*), in LB broth (for *E. coli*) or on agar plates. The plasmids used to transform *S. aureus* strains were maintained in strain RN4220 prior to conducting the experiments.

**Table 1:** Bacterial strains and plasmids used in this study.

Strain or plasmid	Description^a^	Reference or source
**Strains**		
*Escherichia coli*		
DH5α	General molecular cloning	Invitrogen
*Enterococcus faecalis*		
HIP12467	pAM830::Tn*1546*; Van^R^	NARSA^b^ [35]
*Staphylococcus aureus*		
RN4220	Plasmids reservation	NARSA
ATCC12598	Standard strain	ATCC
SJC1200	ATCC12598/pG1546; Van^R^	This study
SJC1205	SJC1200, Δ*sigB*::*spc*; Van^R^, Spc^R^	This study
SJC1206	SJC1205, *rocA*:: *sigB* operon; Van^R^, Spc^R^	This study
**Plasmids**		
pGHL6	*E. coli*/*S. aureus* shuttle vector	[36]
pG1546	pGHL6/Δ*luxAB*::*van* operon	This study
pMAD	Vector for allelic replacement	[18]
pMAsigBD	pMAD/*sigB*::*spc*	This study
pMAsigBC	pMAD/*rocA*::*sigB* operon	This study

a Abbreviations: Van^R^, vancomycin resistant; Spc^R^, spectinomycin resistant.

b Network on Antimicrobial Resistance in *Staphylococcus aureus* (NARSA).

**Table 2:** Primers used in this study.

Primer	Sequence (5′ → 3′)^a^
**Cloning**	
Tn-SphI-F	AAAGCATGCAGGAATGAATTATGCGG
Tn-KpnI-R	CACTTGGTACCTACGGGCGAGTTTC
RsbU-BglII-F	CGGAAGATCTGACTGAAGCTAG
SigB-NcoI-R	GCTCAGGTGAAACTTCCATGGCTGATTTC
SigB-SalI-F	CGTCGACTAAGAAATTACAAGAAGCAGC
SA2151-BamHI-R	TTCTTGACGTGCAATGGGATCCTCAC
SigBop-SalI-F	CGGTCGACTATTGAAAATGACACACCATC
SigBop-NcoI-R	AACCATGGCGTCTATTATATGTATTTTTCAGAG
RocA-BamHI-F	CCTGGATCCACACCTAAGATGTG
RocA-SalI-R	CATGTGAATGGTCGACATTTAAAC
RocA-NcoI-F	CTGTTATGCATATACCATGGTC
RocA-BglII-R	GCATTAGAACAGATCTGAAACAACC
**qRT-PCR**	
sigB-F	TGGCGAAAGAGTCGAAATCAGC
sigB-R	TCAGCGGTTAGTTCATCGCTCAC
asp23-F	AAAATTGCTGGTATCGCTGC
asp23-R	TGTAAACCTTGTCTTTCTTGGT
fnbA-F	ACTGGCGCAGTGAGCGACCA
fnbA-R	GCACTTCTGGCGTTGGCGGT
hla-F	CCTGGCCTTCAGCATTTAAG
hla-R	GGTCCCCAATTTTGATTCAC
dnaA-F	TCCACATGCAGCGAGTTTAG
dnaA-R	GGTGGTCGATCACTCGAAAT

a Incorporated restriction sites are underlined.

The vancomycin-resistant *S. aureus* strain SJC1200 was generated by introducing a vancomycin resistance-carrying plasmid (pG1546) into strain ATCC 12598 as described previously [17]. The *P_R_vanRSP_H_HAXP_Y_vanYP_Z_vanZ* gene cluster (the *van* operon within Tn*1546*) in *E. faecalis* HIP12467 was amplified using the primer pair Tn-SphI-F and Tn-KpnI-R, which contained restriction sites for *Sph*I and *Kpn*I. The 7075-bp PCR product was then cloned into pGHL6 from which the *luxAB* gene was removed by digestion with the same restriction enzymes (New England Biolabs) to generate pG1546. VRSA strains, approved by the Chang Gung University biosafety committee, were used strictly in a P2 level laboratory, and all lab equipment and surfaces were sterilized by bleach and/or autoclaved immediately after the experiments were performed.

Allelic replacement of the *sigB* gene by a spectinomycin cassette (*spc*) was performed by introducing the pMASigBD plasmid into strain SJC1200 to generate the *sigB* mutant strain SJC1205, as described previously [18]. A spectinomycin cassette restricted by *Nco*I and *Sal*I was flanked by the upstream and downstream arms of the PCR product. The 1115-bp upstream arm originating from the 5′ end of the *sigB* was amplified using the primer pair RsbU-BglII-F and SigB-NcoI-R, which contained *Bgl*II and *Nco*I restriction sites, respectively. The 1183-bp downstream arm restricted by *Sal*I and *Bam*HI was amplified using the primer pair SigB-SalI-F and SA2151-BamHI-R from the 3′ end of *sigB*. The constructed upstream arm-*spc*-downstream arm DNA fragment restricted by *Bgl*II and *Bam*HI was then cloned into pMAD, yielding pMASigBD.

Complementation of the *sigB* mutation in strain SJC1205 was performed by inserting the full-length *rsbU* and *sigB* operon (*P_U_rsbUP_V_rsbVWsigB*), including the promoter region, into the *rocA* gene via homologous recombination to generate SJC1206 [19]. The 3277-bp *P_U_rsbUP_V_rsbVWsigB* DNA fragment restricted by *Sal*I and *Nco*I was amplified using the primer pair SigBop-SalI-F and SigBop-NcoI-R and was flanked by parts of the 5′ and 3′ ends of *rocA*. The 484-bp 5′ region was amplified using the primer pair RocA-BamHI-F and RocA-SalI-R, whereas the 489-bp 3′ region was amplified using RocA-NcoI-F and RocA-BglII-R. The 5′ region-*P_U_rsbUP_V_rsbVWsigB*-3′ region DNA fragment was cloned into pMAD, yielding pMAsigBC, which was then used to transform into SJC1205. The *sigB*-complemented colonies were selected using blue-white screening at the non-permissive temperature [18]. The insertion of *rocA* by *P_U_rsbUP_V_rsbVWsigB* was confirmed by PCR and DNA sequencing.

### Detection of gene expression and real-time quantitative reverse transcription PCR (qRT-PCR)

The activity of σ^B^ upon treatment with antibiotics with distinct modes of action was detected using RT-PCR by assaying the transcription of *asp23*, a gene encoding an alkaline shock protein, which is directly activated by σ^B^ [20]. The MICs of the antibiotics used in the present study were determined by an E-test and a microdilution broth method according to CLSI guidelines [21]. The MICs of the antibiotics for strain SJC1200, and the final concentrations used in the qRT-PCR assay are provided in Table 3. Antibiotics were added to bacterial cultures at the indicated concentrations at OD_600_ = 0.6. Antibiotic-treated or untreated bacterial cells were collected and pelleted at a given time and frozen on dry ice immediately. Total RNA was extracted from cell pellets using TRIzol (Invitrogen) followed by RQ1 RNase-free DNase (Promega) treatment to eliminate any remaining DNA. An RT-PCR time course was performed, and the results were visualized using agarose gel electrophoresis to determine the time points for subsequent qRT-PCR analysis. The mRNA levels, including those of *sigB*, *asp23*, *fnbA* and *hla*, upon vancomycin treatment (32 µg/ml) were determined by qRT-PCR with the KAPA™ SYBR® qPCR Kit (Kapa Biosystems) in a Roche LightCycler (LC-32). All samples were tested in triplicate in three independent experiments. The expression levels of different genes were normalized against the *dnaA* expression level. The fold change of each transcript was determined by the 2^−ΔΔCT^ method compared with the untreated cells [22].

**Table 3:** MICs of antibiotics for strain SJC1200 and the final concentrations treated in qRT-PCR for all of the tested strains.

	Antibiotic^a^				
	Van	Amp	Cm	Cip	SXT
MIC^b^ (µg/ml)	>256	32	64	0.8	2
Treated concentration^c^ (µg/ml)	16 or 32	12	16	0.25	0.8

a Abbreviations: Van: vancomycin; Amp: ampicillin; Cm: chloramphenicol; Cip: ciprofloxacin; SXT: sulfamethoxazole/trimethoprim.

b The MICs of antibiotics for strains SJC1205 and 1206 were the same as for SJC1200.

c The concentrations used were based on the therapeutic levels except for SXT.

### Contact hemolysis

Contact hemolysis was performed as described elsewhere [23]. The bacteria were cultured overnight, diluted to OD_600_  = 0.1 and subcultured in 20 ml of fresh LB Broth for about three hours at 37°C at which point the culture was in log phase (OD_600_ = 0.6∼0.8). The bacterial cultures were collected by centrifugation at 10,000×*g* for 10 minutes at 4°C and resuspended in 20 ml of cold PBS. Sheep blood was washed three times in PBS by centrifugation at 1,500×*g* for five minutes at 4°C and diluted to a final concentration of 8%. A 100 µl aliquot of bacterial culture was mixed with 900 µl of 8% red blood cells (10-fold dilution of bacteria) in a 1.5 ml centrifuge tube and incubated at 37°C for three hours. Finally, the mixtures were centrifuged at 1,500×*g* for 10 min at 4°C, and the OD_450_ was measured using a Novaspec II spectrophotometer (Pharmacia Biotech). The positive control sample contained erythrocytes that had been lysed by SDS. One unit of hemolytic activity was defined as half of the total erythrocytes lysed by hemolysin relative to the positive control at OD_450_, followed by multiplication by the dilution factor to obtain the final hemolytic unit.

### Cytotoxicity assay

The cytotoxicity assay was performed by evaluating the cell viability of BEAS-2B cells (human bronchial epithelial cells) co-cultured with *S. aureus* in the presence of different concentrations of vancomycin. BEAS-2B cells were maintained in RPMI-1640 medium (Gibco BRL) supplemented with 10% fetal bovine serum, 10% glycine and 1% penicillin and streptomycin at 37°C in a humidified atmosphere of air and 5% CO_2_. Cell viability tests were performed using the MTT assay with the cell proliferation reagent MTT (3-[4,5-dimethylthiazol-2-yl]-2,5-diphenyl tetrazolium bromide; Sigma), as described previously [24]. The MTT tetrazolium ring is cleaved only by active mitochondria, yielding purple formazan crystals whose amount directly correlates with the viable cell count. Cells were inoculated with different *S. aureus* strains (multiplicity of infection of 100) in the presence of different concentrations of vancomycin. In a parallel experiment, polyclonal fibronectin antibodies (5 µg/ml; Abcam) were added to block the interaction between fibronectin-binding proteins and cells. After six hours of exposure, 10 µl of a 5 mg/ml MTT solution was added into each well, and the plates were incubated at 37°C for 2.5 hours. The purple formazan crystals were dissolved by adding 100 µl of MTT solubilization solution (Sigma), and the absorbance at A_570_ was spectrophotometrically measured with a reference wavelength of A_690_. The results were expressed as the percent absorbance of the vancomycin-treated cultures versus the untreated control cultures. Three wells per dose were counted in three independent experiments.

### Statistical analysis

A Student's *t*-test was used to analyze the experimental data and to compare means. *P* values of less than 0.05 were considered statistically significant.

## Results

### Effect of antibiotic treatment on the expression of σ^B^

**Figure 1:**
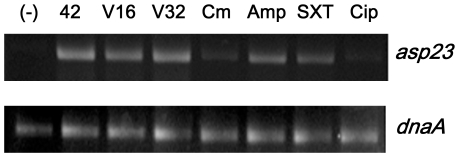
Effects of different antibiotics with distinct modes of action on *asp23* transcription. (-): sample without treatment (negative control); 42: heat shock at 42°C for five minutes (positive control); V16 and V32: vancomycin at the concentrations of 16 and 3 µg/ml, respectively.

Strain SJC1200 was treated with antibiotics with distinct modes of action at sub-inhibitory concentrations as shown in Table 3. Bacterial growth was assessed by monitoring the growth curve spectrophotometrically at OD_600_. The plateau level was not affected by antibiotic treatment, but the stationary phase was reached later than in the drug-free condition (data not shown). The expression of *asp23*, which is directly activated by σ^B^, was increased at 10 minutes post-treatment with ampicillin, vancomycin, and SXT (Fig. 1).

**Figure 2:**
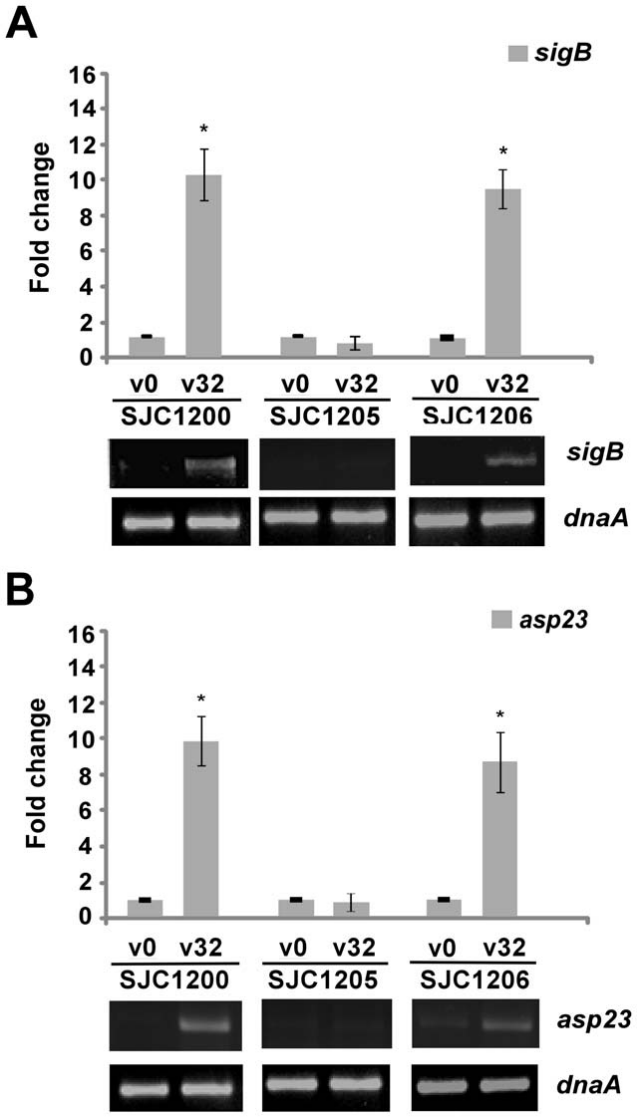
Expression of *sigB* upon vancomycin treatment. Changes in the transcription levels of the (A) *sigB* and (B) *asp23* genes upon vancomycin treatment determined using qRT-PCR. Representative agarose gel electrophoresis images of RT-PCR assays are shown below the bar chart. V0 and V32: 0 and 32 µg/ml vancomycin, respectively. * *P*<0.05 compared to the untreated control cells.

We next focused on the effects of vancomycin treatment on the expression of *sigB* using real-time qRT-PCR. It had been recommended that the appropriate therapeutic range of vancomycin concentrations in the serum is 15 to 20 µg/ml for through levels and 20 to 40 µg/ml for peak levels [25]. A recent study also indicated that 74% of heteroresistant VISA (hVISA) strains and 15% of wild-type *S. aureus* strains were tolerant to the effects of vancomycin (minimum bactericidal concentration of ≧ 32 µg/ml) [26]. Therefore, the maximum concentration of vancomycin used in our experiments was 32 µg/ml. Over the time course examined, the RT-PCR results showed that the peak expression of *sigB* and *asp23* was five and 10 minutes after vancomycin treatment, respectively, and samples were collected for subsequent qRT-PCR assays at these time points (Fig. S1). The results shown in Fig. 2A indicate that the expression of *sigB* was significantly increased (10.2-fold) five minutes after vancomycin treatment. The transcription of *sigB* was not detected in SJC1205 (*sigB*-deficient strain) and was restored in SJC1206 (*sigB*-complemented strain). To further confirm the activation of σ^B^, *asp23* mRNA levels were quantified. Consistent with the expression of *sigB*, the expression of *asp23* was significantly increased (9.8-fold) in strain SJC1200 10 minutes after vancomycin treatment, suggesting that σ^B^ was activated (Fig. 2B). The activation of *asp23* was abolished in strain SJC1205 and was restored in SJC1206. Vancomycin-stimulated σ^B^ activation was also observed in VRSA strains derived from the strain COL and ATCC 49476 genetic backgrounds and in the VISA strain Mu50 at the concentrations of 32 and 4 µg/ml, respectively (data not shown).

### Effect of vancomycin on the expression of virulence-associated genes

**Figure 3:**
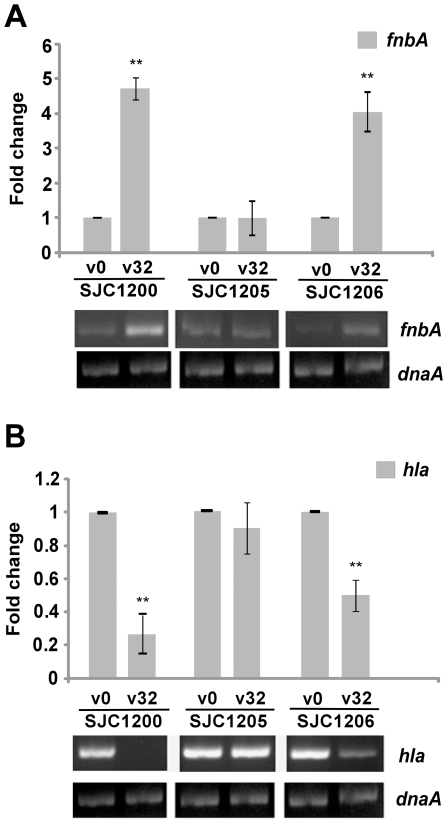
Expression of virulence genes upon vancomycin treatment. Changes in the transcription levels of (A) *fnbA* and (B) *hla* genes upon vancomycin treatment determined using qRT-PCR. Representative agarose gel electrophoresis images of RT-PCR assays are shown below the bar chart. * *P*<0.05 and ** *P*<0.005 compared to the untreated control cells.

The transcription of *hla* and *fnbA* mRNA over time in response to vancomycin treatment was investigated using RT-PCR. The greatest difference in the expression levels of *fnbA* and *hla* between drug-treated and untreated cells was observed one hour after vancomycin treatment, and this time point was used for further qRT-PCR assays (Fig. S2). A significant increase in the transcription level of *fnbA* (4.8-fold) was observed one hour after treatment of vancomycin in strain SJC1200. The increased *fnbA* expression was abolished in strain SJC1205 and was restored in SJC1206 (Fig. 3A). However, *hla* expression was significantly decreased (0.2-fold) after one hour of vancomycin treatment. The decreased *hla* expression was not observed in strain SJC1205 and was restored in SJC1206 (Fig. 3B).

### Effect of vancomycin on the hemolytic activity

**Figure 4:**
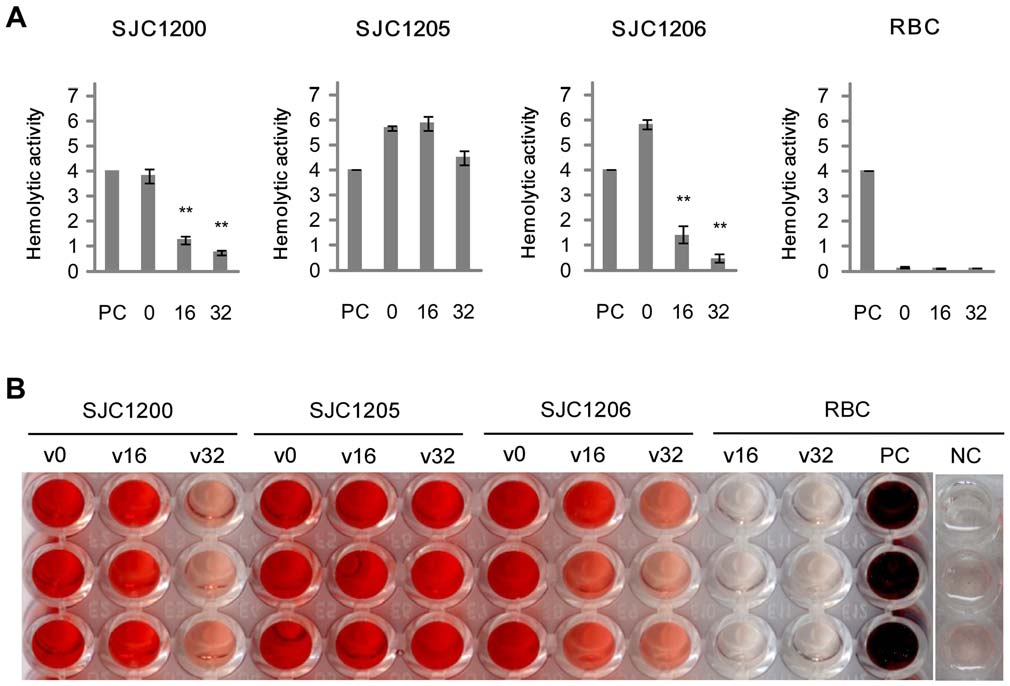
Effect of vancomycin on hemolytic activity. (A) Quantitative hemolytic unit. (B) Hemolysis was observed in a flat-bottom 96-well microtiter plate. Numerals shown on the figure represent the vancomycin concentration (µg/ml). PC: positive control; NC: negative control (erythrocyte suspensions without bacterial inoculation). ** *P*<0.005 compared to the untreated control cells.

The decreased *hla* expression suggests that the hemolytic activity of *S. aureus* is impaired following vancomycin treatment. A hemolytic assay measuring contact hemolysis was performed to evaluate the effect of vancomycin-induced σ^B^ activity on hemolysis. As expected, vancomycin treatment significantly suppressed hemolysis by strains SJC1200 and SJC1206 and mildly reduced hemolysis in the *sigB*-deficient strain SJC1205 (Fig. 4A and 4B).

### Effect of vancomycin-induced σ^B^ activity on cytotoxicity

**Figure 5:**
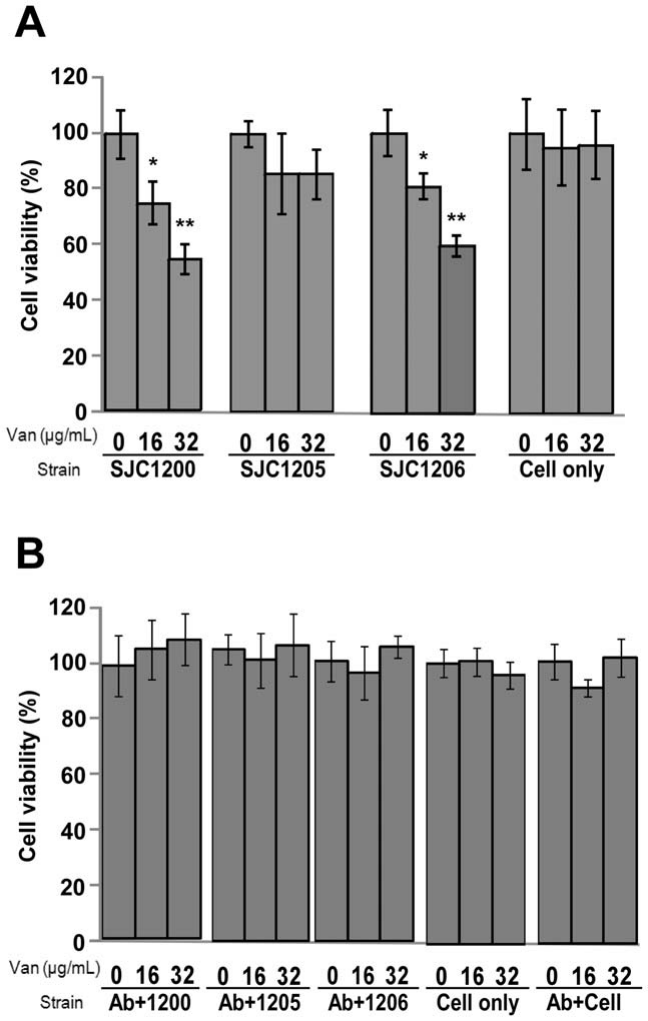
Effect of vancomycin-treated VRSA on the cytotoxicity. Cytotoxicity toward BEAS-2B cells inoculated with *S. aureus* strains in the presence of different concentrations of vancomycin (A) without or (B) with polyclonal fibronectin antibodies. “Cell only” indicates cells cultured without bacteria to evaluate the cytotoxicity of vancomycin. “Ab+cell” indicates cells cultured with polyclonal fibronectin antibodies to evaluate the cytotoxicity of the antibodies. * *P*<0.05 and ** *P*<0.005 compared to the untreated control cells.

The pathogenicity of *S. aureus* involves the net expression of different exotoxins and cell wall components. Whether the vancomycin-induced σ^B^ activity affected *S. aureus* pathogenicity was investigated by a cytotoxicity assay. Because *S. aureus* is a major pathogen of the airway, cytotoxicity was evaluated in BEAS-2B cells using the MTT assay. When strains SJC1200 and SJC1206 were challenged with vancomycin, the cell viability was significantly decreased in a dose-dependent manner (Fig. 5A). The viability of BEAS-2B cells co-cultured with SJC1200 decreased to 74% (*P*<0.05) upon treatment with vancomycin at 16 µg/ml and further decreased to 57% (*P*<0.005) at 32 µg/ml. No considerable cytotoxicity was detected by challenging strain SJC1205 under the same conditions.

Vancomycin-enhanced *fnbA* expression suggests that the increased cytotoxicity may result from an increase in bacterial attachment to cells. A parallel cytotoxicity experiment was performed in which the target of fibronectin-binding proteins, fibronectin was blocked using antibodies. Vancomycin-enhanced cytotoxicity was abolished among all of the three strains in the presence of fibronectin antibodies (Fig. 5B). Cytotoxicity was not observed in control cells cultured with different concentrations of vancomycin or polyclonal fibronectin antibodies in the absence of bacteria (Fig. 5A and 5B).

## Discussion

In the present study, we demonstrated that σ^B^ was activated when *S. aureus* was exposed to sub-inhibitory concentrations of antibiotics that target the cell wall (ampicillin and vancomycin) or SXT. In particular, we focused on the effects of vancomycin treatment on a VRSA strain. We demonstrated that vancomycin-stimulated σ^B^ is involved in the expression of virulence-associated genes and the increase in cytotoxicity. A recent study also indicated that the alternative sigma factor σ^E^, which is responsible for the surface stress response, is activated by vancomycin in *Mycobacterium tuberculosis* [27].

We demonstrated that only the cell-wall active antibiotics and SXT acted as environmental stressors that induced σ^B^ transcription (Fig. 1). It has been reported that treatment with protein-suppressing agents may not cause significant differences in the expression of virulence factors in *S. aureus* [14], [15]. We propose that cell wall-targeting antibiotics and SXT trigger unknown receptors that induce σ^B^ activity. Activation of σ^B^ by vancomycin was observed in the SJC1200 strain (ATCC 12598 derivative), in the VISA strain Mu50, and in all of the tested VRSA strains derived from the strain COL and the ATCC 49476 genetic backgrounds (data not shown), suggesting that antibiotic-induced σ^B^ activity occurs frequently. Strain 12598 was chosen for subsequent studies because of its strong hemolysis and cytotoxicity in our previous study. DNA sequence analysis indicated that there were no mutations in the *sigB* operon of strain 12598 (data not shown).

Cheung and colleagues demonstrated the hyper-production of α-hemolysin in a *sigB* mutant, implying a negative effect of σ^B^ on *hla* expression [28]. Restoring σ^B^ activity to a σ^B^-impaired strain decreased Hla expression [8]. Consistent with these findings, vancomycin-activated σ^B^ significantly suppressed *hla* expression, thereby reducing hemolysis. In addition to exotoxins such as hemolysins, cell wall-associated proteins, such as fibronectin-binding proteins (FnBPs), are important virulence determinants. The surface levels of FnBP were lower in a *sigB*-deficient strain, suggesting that σ^B^ regulates FnBP expression [29]. It has been shown that the increased expression of FnBPs is related to the diminished expression of Hla [30], [31]. Our results are consistent with previous findings in that increased *fnbA* transcription was associated with decreased *hla*. Thus, the interplay between σ^B^ activation and the expression of *hla* and *fnbA* is associated with vancomycin treatment. Although the expression of many virulence factors in *S. aureus* is regulated by the *agr*-mediated temporal and cell density-dependent regulatory pathways [9], vancomycin treatment still had a significant and immediate effect on the expression levels of virulence factors during the exponential growth phase (Fig. S2). The rapid activation of σ^B^ in response to vancomycin stress may force virulence expression through cell density-independent pathways. Generally speaking, the expression of *fnbA* was increased over time in *sigB* ^+^ strains with or without vancomycin treatment, whereas *hla* was increased up to two hours after vancomycin treatment (Fig. S2).

A striking finding of the present study is that σ^B^ was activated by cell wall-targeting antibiotics and SXT in drug-resistant *S. aureus* strains. This sigma factor may mediate the antibiotic-activated signal, as well as other environmental stresses, to downstream virulence determinants, leading to worse outcomes in the clinical environment. The results from the cytotoxicity assays reported herein reflect this concern. A significant decrease in cell viability upon administration of increased concentrations of vancomycin in the presence of drug-resistant *S. aureus* was observed (Fig. 5). Cytotoxicity induced by vancomycin was not observed when BEAS-2B cells were co-cultured with the *sigB*-deficient *S. aureus* strain SJC1205, suggesting the key role of σ^B^ in responding to antibiotic stress. The rapid increase in the prevalence of multidrug-resistant pathogenic bacteria and the appearance of resistant strains following continuous selective pressure suggest that improper antibiotic use may occur in the clinical environment. Such improper use may be the result of unsuitable initial antibiotic treatment, the development of drug-resistant strains during long-term selective pressure or inappropriate treatment following misdiagnosis. Regardless, antibiotic-enhanced pathogenicity makes subsequent treatment much more difficult. A recent study indicated that the highest mean steady-state concentrations of vancomycin for continuous and intermittent infusion regimens were 24.88±12.75 and 55.02±17.36 µg/ml, respectively, whereas the lowest concentrations were 19.89±10.15 and 12.43±12.86 µg/ml, respectively, in serum [32]. Concentrations are expected to be even lower in tissues. As a result, VISA strains could survive and enhance pathogenicity. Our results indicate that σ^B^ was activated in strain Mu50 after treatment with a lower concentration of vancomycin (4 µg/ml, data not shown), suggesting that the expression of σ^B^-associated virulence genes might be altered.

The model strain used in the present study was derived from the ATCC 12598 (Cowan I) genetic background, which has been recognized as a protein A-overproducing and archetypal adherent strain. This strain is also known to lack a variety of exotoxins such as most staphylococcal enterotoxins, exfoliative toxins and toxic shock syndrome toxin 1 [33]. Strain ATCC 12598 is often used as a control strain in determining the invasive capacity and cytotoxicity of other strains. It has been demonstrated that FnBP expression is highly associated with the invasion of host cells, particularly during the study of ATCC 12598 [34]. Following vancomycin treatment, no obvious increase in exoprotein secretion by strain SJC1200 was observed by SDS PAGE analysis (data not shown), but there was a decrease in Hla expression. This result suggests that the cytotoxicity caused by SJC1200 was mainly due to the σ^B^-activated cell wall components, possibly FnBPs, upon vancomycin treatment. A parallel study was performed using a VRSA strain derived from the strain COL genetic background, and similar cytotoxicity results were observed. Based on many studies, *fnbA* could have some role in invasion, but the overall cytotoxicity (cell invasion and damage) is governed by various toxins. Because SDS-PAGE is not a sensitive determinant of protein secretion, the expression of trace exoproteins stimulated by vancomycin may not be observable using a gel. In addition, BEAS-2B cells may not be the best model for analyzing virulence, particularly because they are less sensitive to Hla. However, the blockage of fibronectin by antibodies leading to subsequent reduction of cytotoxicity implies that vancomycin-enhanced bacterial attachment plays an important role in pathogenesis, at least in bronchial epithelial cells (Fig. 5B). The role of toxins underlying cytotoxicity after bacterial attachment and invasion needs to be investigated. Nevertheless, we propose that other σ^B^-regulated exotoxins are overexpressed upon antibiotic treatment in other drug-resistant strains.

In conclusion, we hypothesize that sub-MICs of antibiotics may act as environmental stresses to activate the stress response sigma factor, σ^B^. Although it has been reported that a number of virulence-associated genes are regulated by σ^B^, the complexity of the downstream global regulatory pathways shows the diversity of virulence gene expression levels, not only within a single strain but also among different lineages. The pathogenicity of *S. aureus* will be the net effect of virulence-associated regulatory pathways and strain dependence. Although we cannot give a definitive warning about the antibiotic-triggered σ^B^-associated virulence factor expression in pathogenic *S. aureus* because of strain variation, the risk should be taken into consideration.

## Supporting Information

Figure S1 **Evaluation of the expression levels of** ***sigB*** **and** ***asp23*** **over time using RT-PCR.** The agarose gel electrophoresis image shows the time course of the expression of *sigB* and *asp23* in strains SJC1200, SJC1205, and SJC1206 without (V0) or with (V32) vancomycin treatment using RT-PCR.
(TIF)

Figure S2 **Evaluation of the expression levels of** ***fnbA*** **and** ***hla*** **over time using RT-PCR.** The agarose gel electrophoresis image shows the time course of the expression of *fnbA* and *hla* in strains SJC1200, SJC1205, and SJC1206 without (V0) or with (V32) vancomycin treatment using RT-PCR.
(TIF)
