# Supplementary figures and images for: Vancomycin Activates σB in Vancomycin-Resistant Staphylococcus aureus Resulting in the Enhancement of Cytotoxicity
Source: PLoS One. 2011 Sep 2;6(9):e24472. doi: 10.1371/journal.pone.0024472 (PMC3166330; doi:10.1371/journal.pone.0024472)

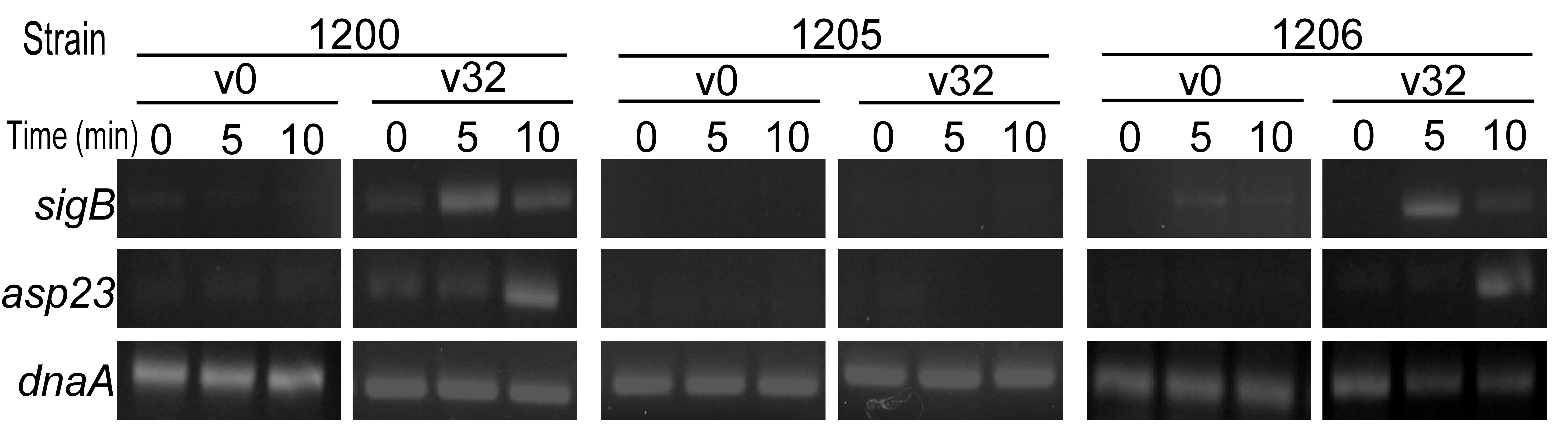

Supplement: Figure S1 — Evaluation of the expression levels of sigB and asp23 over time using RT-PCR. The agarose gel electrophoresis image shows the time course of the expression of sigB and asp23 in strains SJC1200, SJC1205, and SJC1206 without (V0) or with (V32) vancomycin treatment using RT-PCR. (TIF) [file pone.0024472.s001.tif]

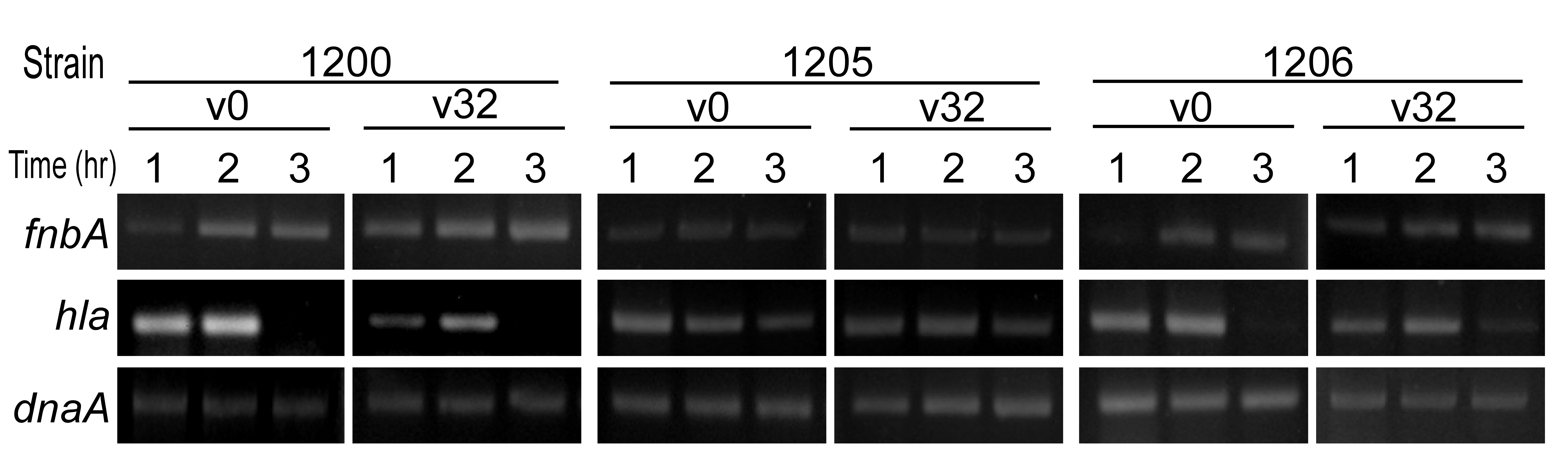

Supplement: Figure S2 — Evaluation of the expression levels of fnbA and hla over time using RT-PCR. The agarose gel electrophoresis image shows the time course of the expression of fnbA and hla in strains SJC1200, SJC1205, and SJC1206 without (V0) or with (V32) vancomycin treatment using RT-PCR. (TIF) [file pone.0024472.s002.tif]
